# Risk factors associated with early mortality after recovery from severe listeriosis: a multicentre 17-year longitudinal study

**DOI:** 10.1007/s15010-022-01872-1

**Published:** 2022-06-26

**Authors:** Mario Rivera-Izquierdo, María Dolores Galicia-García, Antonio Jesús Láinez-Ramos-Bossini, Pablo Redruello-Guerrero, Nicolás Francisco Fernández-Martínez

**Affiliations:** 1 Department of Preventive Medicine and Public Health, University of Granada, Avenida de la Investigación 11, 18016 Granada, Spain; 2 Section of Epidemiology, Provincial Health Delegation of Granada, Granada, Spain; 3 Service of Preventive Medicine and Public Health, Hospital Universitario San Cecilio, Granada, Spain; 4 Instituto Biosanitario de Granada ibs.GRANADA, Granada, Spain; 5 Hospital Universitario Virgen de Las Nieves, Granada, Spain; 6 Faculty of Medicine, University of Granada, Granada, Spain; 7 Unidad de Gestión Clínica Interniveles de Prevención, Promoción y Vigilancia de La Salud, Hospital Universitario Reina Sofía, Córdoba, Spain; 8 Preventive Medicine and Public Health Research Group, Maimonides Institute for Research in Biomedicine of Cordoba (IMIBIC), 14004 Córdoba, Spain

**Keywords:** Listeria monocytogenes, Acute infection, Comorbidities, Survival, Prognosis, Hospitalisation

## Abstract

**Background:**

Listeriosis presents high rates of mortality but prognostic factors for early prevention are not well established. The aim of this study was to analyse factors associated with in-hospital and early mortality of adults after recovery from severe infection caused by *Listeria monocytogenes*.

**Methods:**

All cases of listeriosis notified in the province of Granada from January 2005 to December 2021, including 9 centres, were included. Only laboratory confirmed non-neonatal cases were considered. Follow-up was conducted by accessing medical records and epidemiological data. Bivariate and multivariate analyses were conducted to detect potential risk factors associated to in-hospital mortality, 1-year, and 5-year early death after recovery. Multivariate Cox regression models were performed. A total of 206 patients were identified.

**Results:**

The mean age was 62.6 years (sd, 18.8). A high frequency of comorbidities (88.3%) was observed, and 42 patients (20.4%) died during hospitalisation. Of the patients who recovered from acute infection, 26 (15.9%) died during the following year and 47 (28.7%) died during the following 5 years. The main factors associated with early mortality after recovery were age (HR: 1.03; 95% CI 1.02–1.07), diabetes mellitus (HR 1.86, 95% CI 1.01–3.44), chronic kidney disease (HR 3.96, 95% CI 1.87–8.38), liver disease (HR 3.62, 95% CI 1.64–8.51), and cancer (HR 3.76, 95% CI 1.90–7.46).

**Conclusion:**

Listeriosis is associated with high early post-recovery mortality. Our study describes the main prognostic factors, which may help to improve preventive follow-up strategies of adults with severe listeriosis.

## Introduction

*Listeria monocytogenes* is a ubiquitous motile facultative anaerobic Gram-positive bacillus (bacterial species) with the ability to persist under adverse conditions [1]. Generally, the disease caused by this agent (listeriosis) in adults selectively affects pregnant women, patients at extreme ages or patients with various types of immunosuppression [2]. Ingestion of contaminated food is the most common source of infection, which usually has a limited course in immunocompetent patients. However, listeriosis may present in an invasive life-threatening form in high-risk groups, with high mortality rates [2]. Invasive disease usually presents as meningitis, meningoencephalitis or primary bacteraemia that can lead to sepsis. Most studies tend to separate severe listeriosis into two groups based on clinical presentation: bacteraemic clinical course (e.g. bacteraemia or sepsis) and neurolisteriosis (e.g. meningitis, meningoencephalitis or rhombencephalitis [3].

In the United States, high hospitalisation and mortality rates due to listeriosis have been reported [3–6]. In Europe, listeriosis is one of the most serious foodborne infections [7]. The incidence of this disease has gradually increased in Europe in recent years, with the exception of 2020, probably due to the COVID-19 pandemic. Spain, together with France and Poland, are the countries reporting the highest number of cases requiring hospitalisation [8]. According to hospital data, listeriosis has been on an upward trend in Spain since 1997 [9].

Several factors associated with severe forms (i.e. those requiring hospitalisation or causing death) of listeriosis have been suggested, including severe immunosuppression, concomitant presence of serious conditions (e.g. cancer), seasonality and low neutrophil/lymphocyte ratio [10]. However, to date, factors associated with early mortality after resolution of active infection remain unknown.

Our hypothesis is that listeriosis is a marker for a group of patients with particular vulnerability, whose short-term prognostic outcomes are poor, and who can be identified to improve follow-up prevention.

The aim of this study was to analyse (1) factors associated with in-hospital mortality due to listeriosis and (2) factors associated with early mortality after resolution of infection, in a cohort of confirmed cases in Granada, Spain.

## Methods

The *Strengthening the Results of Observational studies in Epidemiology* (STROBE) guideline [11] was followed for the presentation of the results of this study, in accordance with the recommendations of the EQUATOR guidelines.

### Study design and setting

We conducted a longitudinal observational cohort study using a retrospective case series that compiled all cases of listeriosis notified in the province of Granada, Spain, through the electronic application (RedAlerta) of the Andalusian Epidemiological Surveillance System (the first notified cases of listeriosis in Granada started in 2005) to 2021. Only confirmed cases (i.e. those with a confirmatory laboratory test identifying *L. monocytogenes* in a clinical sample) were included. All cases were followed up retrospectively until December 31, 2021.

### Data sources and variables

The main sources of data for this study were the Andalusian Epidemiological Surveillance System, which allowed the identification of all notified cases of listeriosis, and the electronic medical records of the cases selected. Hospitalisation reports of cases that required hospital admission were accessed, and the primary care medical records were consulted to collect relevant sociodemographic and clinical information. The main variables considered in this study were:

- Sociodemographic variables: age, sex, and year of diagnosis.
- Epidemiological and environmental variables: hospital or primary care centre.
- Dates: date of case report, date of onset of symptoms, date of admission, date of discharge, date of death.
- Previous comorbidities, immunosuppression (we considered in this group those patients that showed immunosuppression in blood tests, or those under immunosuppressing treatments), and pregnancy.
- Clinical presentation of the disease: meningitis, meningoencephalitis, rhombencephalitis, bacteraemia (defined as the identification of *L. monocytogenes* in blood tests without associated septic syndrome), sepsis (which included septic syndrome or septic shock), gastroenteritis, device-associated infection, or others.
- Follow-up variables: cause of death after resolution of active infection (cancer disease, infection or other causes).

The main outcomes considered were (1) in-hospital death during active infection (2) death during the first year after resolution of active infection, and (3) death during the first 5 years after resolution of active infection.

### Statistical analyses

First, a descriptive analysis was performed to characterise the entire sample. Absolute frequency (*n*) and relative frequency (%) were used to describe qualitative variables and mean (*x*) and standard deviation (sd) to describe quantitative variables. This analysis was stratified by in-hospital mortality and bivariate analyses were applied to identify risk factors for mortality during hospitalisation. For possible quantitative risk factors, Student's t tests were applied, and for qualitative risk factors, chi-square tests were applied. Next, we focussed on the sample of patients who survived acute infection. This subgroup was evaluated and stratified by the development of our main outcomes of interest, namely 1-year and 5-year mortality after resolution of infection. To identify the main variables associated with mortality after resolution, bivariate analyses were performed. Once the potential risk factors were identified by bivariate analysis, a multivariate analysis was performed. For this purpose, we designed logistic regression models using our three outcomes of interest as dependent variables. Covariates included sex, age, previous comorbidities, year of diagnosis, and clinical presentation of listeriosis. Finally, survival analyses were conducted using the time after clinical recovery of acute listeriosis and were graphically assessed using Kaplan–Meier estimates. Multivariate Cox proportional hazards models were designed. Adjusted hazard ratios were calculated. The conditions of application for Cox regression models were verified and validation of the final models was assessed. Foe each variable, we used Schoenfield residuals to verify the proportionality of hazards. Covariates included the relevant sociodemographic and clinical variables detected in bivariate analyses as potential confounders.

All analyses were performed using the free software R (R Foundation for Statistical Computing, Vienna, Austria; https://www.R-project.org/).

### Ethical considerations

Our study complied with all ethical standards for observational studies in humans and the recommendations stated in the Declaration of Helsinki. We used an anonymized database for all analyses, and all potentially identifying variables were removed. Our study was approved by the Provincial Ethics Committee of Granada (code EML_040222).

## Results

**Fig. 1:**
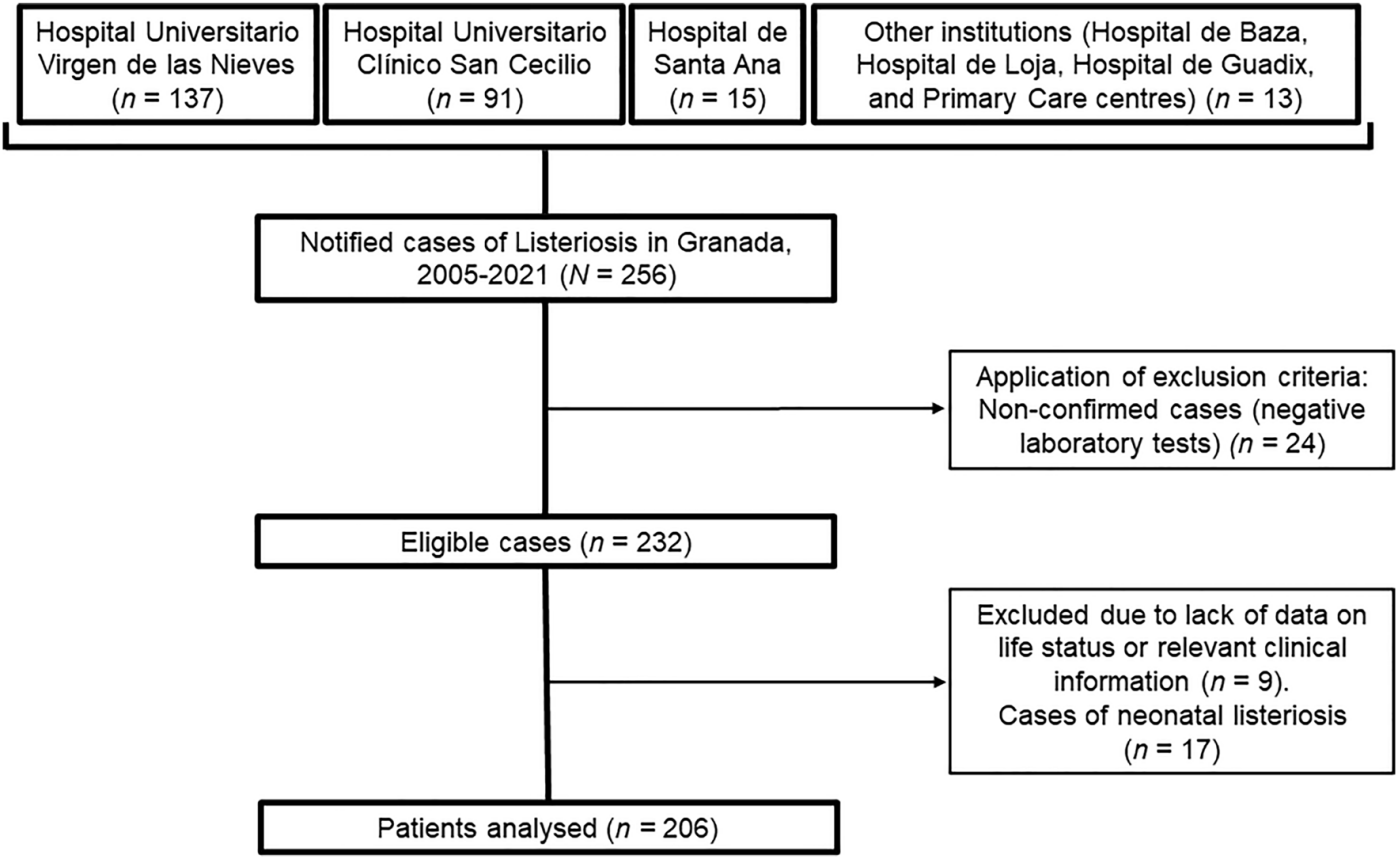
Flow chart of the selection of the sample

The sample of our study consisted of 206 adult patients with confirmed diagnosis of listeriosis in the province of Granada from 2005 to 2021. Figure 1 shows the flow diagram describing how the sample was obtained.

A total of 190 patients (92.2%) required hospital admission due to listeriosis, 56 (29.5%) required admission to the intensive care unit, and 42 (20.4%) died during hospitalisation. Table 1 shows the main sociodemographic and clinical characteristics of the adult sample, stratified by in-hospital mortality.

**Table 1:** Main characteristics of adult listeriosis cases stratified by in-hospital mortality

Characteristic	Total sample	In-hospital death	Surviving patients	*P*-value^1^
Total	206	42 (20.4)	164 (79.6)	–
Sex, *n* (%)				0.096
Women	82 (39.8)	12 (14.6)	70 (85.8)	
Men	124 (60.2)	30 (24.2)	94 (75.8)	
Age, x (sd)	62.6 (18.8)	72.0 (16.3)	60.1 (18.7)	< 0.001*
Age, *n* (%)				0.020*
< 40 years	30 (14.6)	2 (6.7)	28 (93.3)	
41–50 years	25 (12.1)	1 (2.4)	24 (96.0)	
51–60 years	27 (13.1)	5 (18.5)	22 (81.5)	
61–70 years	41 (19.9)	9 (22.0)	32 (78.0)	
71–80 years	42 (20.4)	11 (26.2)	31 (73.8)	
> 80 years	41 (19.9)	14 (34.1)	27 (65.9)	
Year of diagnosis, *n* (%)				0.501
2005–2013	79 (38.3)	18 (22.8)	61 (77.2)	
2014–2021	127 (61.7)	24 (18.9)	103 (81.1)	
Comorbidities				
None	24 (11.7)	1 (4.2)	23 (95.8)	0.036*
Diabetes mellitus	55 (26.7)	13 (23.6)	42 (76.4)	0.485
Hypertension	77 (37.4)	21 (27.3)	56 (72.7)	0.058
COPD	18 (8.7)	7 (38.9)	11 (61.1)	0.041*
Asthma	8 (3.9)	2 (25.0)	6 (75.0)	0.741
OAHSD	8 (3.9)	1 (12.5)	7 (87.5)	0.697
Chronic kidney disease	29 (14.1)	11 (37.9)	18 (62.1)	0.011*
Heart failure	14 (6.8)	7 (50.0)	7 (50.0)	0.010*
Ischaemic cardiopathy	23 (11.2)	7 (30.4)	16 (69.6)	0.269
Arrythmia	19 (9.2)	6 (31.6)	13 (68.4)	0.232
Valvopathy	8 (3.9)	2 (25.0)	6 (75.0)	0.741
Alcoholism	18 (8.7)	5 (27.8)	13 (72.2)	0.415
Liver disease	20 (9.7)	1 (5.0)	19 (95.0)	0.084
Cancer	48 (23.3)	15 (31.3)	33 (68.8)	0.033*
Metastasis	14 (6.8)	6 (42.9)	8 (57.1)	0.042*
Chemotherapy	11 (5.3)	4 (36.4)	7 (63.6)	0.240
HIV infection	8 (3.9)	1 (12.5)	7 (87.5)	0.697
Immunosuppression	32 (15.5)	5 (15.6)	27 (84.4)	0.467
Cognitive impairment	7 (3.4)	2 (28.6)	5 (71.4)	0.633
Morbid obesity	11 (5.3)	0 (0.0)	11 (100.0)	0.125
Autoimmune disease	14 (6.8)	0 (0.0)	14 (100.0)	0.078
Pregnancy	13 (6.3)	0 (0.0)	13 (100.0)	0.075
Clinical presentation of listeriosis				
Meningitis	63 (30.6)	13 (20.6)	50 (79.4)	0.954
Meningoencephalitis	26 (12.6)	5 (19.2)	21 (80.8)	0.875
Rhombencephalitis	20 (9.7)	4 (20.0)	16 (80.0)	0.964
Sepsis	24 (11.7)	10 (41.7)	14 (58.3)	0.010*
Bacteraemia	53 (25.7)	9 (17.0)	44 (83.0)	0.475
Gastroenteritis	10 (4.9)	0 (0.0)	10 (100.0)	0.128
Device-associated infection	6 (2.9)	0 (0.0)	6 (100.0)	0.350
Other	4 (1.9)	1 (25.0)	3 (75.0)	1.000

*COPD* Chronic obstructive pulmonary disease, *HIV* Human Immunodeficiency virus, *OAHSD* Obstructive apnoea–hypopnea sleep syndrome

^1^*P*-value of the T test for quantitative variables (age), and of the chi-square test for qualitative variables or Fisher's exact test when the conditions for application of the chi-square test were not met

^*^p < 0.05

**Fig. 2:**
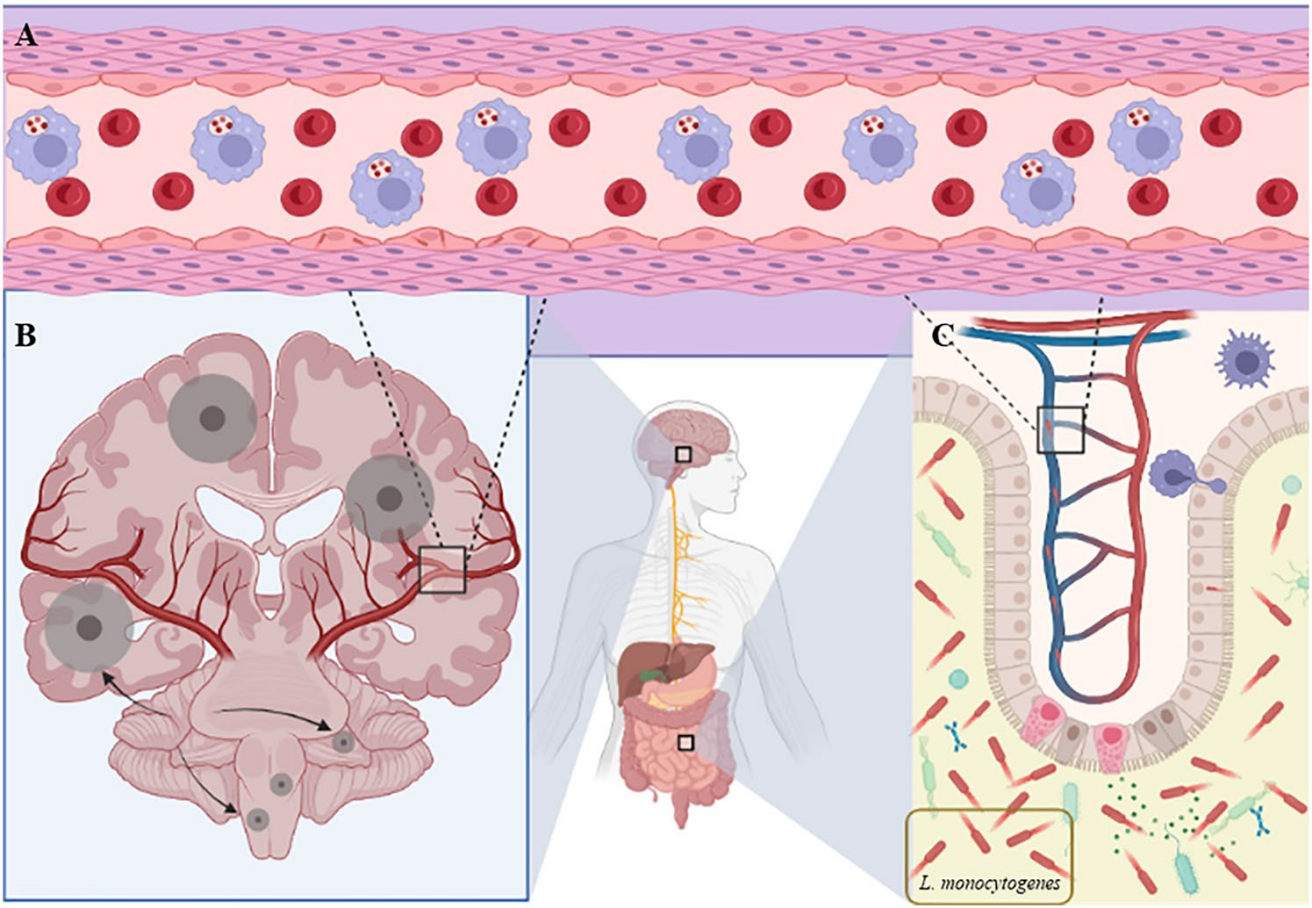
Most frequent clinical manifestations of listeriosis. **A** Blood dissemination of *L. monocytogenes* inside the macrophages. **B** Neurolisteriosis. The arrows show the ring-enhancing lesions affecting the encephalon and rhombencephalon. **C** Gastroenteritis. The bacteria affect intracellularly and provoke the release of cytokines intro the intestinal lumen

The mean age of patients with listeriosis was 62.6 years (standard deviation 18.8), with 60.2% of them being men. A total of 88.3% of the infected patients had some relevant comorbidity. The most frequent comorbidities were hypertension (37.4%), diabetes mellitus (26.7%), cancer (23.3%), immunosuppression (15.5%) and chronic kidney disease (14.1%). Regarding the clinical presentation of listeriosis, the most frequent were meningitis (30.6%), bacteraemia (25.7%), meningoencephalitis (12.6%), sepsis (11.7%), rhombencephalitis (9.8%), gastroenteritis (4.9%) and device-associated infection (2.9%). Therefore, bacteraemic (whether bacteraemia or sepsis), neurolisteriosis and gastroenteritis were the most frequent clinical manifestations (Fig. 2).

The main factors associated with in-hospital mortality in bivariate analyses were advanced age (*p* < 0.001), especially in patients > 80 years (*p* = 0.020), and the presence

of chronic obstructive pulmonary disease (*p* = 0.041), chronic kidney disease (*p* = 0.011), heart failure (*p* = 0.010), cancer (*p* = 0.033), metastasis (*p* = 0.042) and, sepsis as a clinical form of listeriosis (p = 0.010). These variables confirmed the profile of the patient most vulnerable to in-hospital death after admission for listeriosis. The absence of comorbidities (*p* = 0.036) acted as a protective factor, and there was a non-significant trend towards higher mortality in men.

Patients who survived hospitalisation (*n* = 164) were followed up to detect early mortality at 1 and 5 years, respectively. Table 2 shows the main characteristics of the sample of adult survivors stratified by early mortality after discharge. Twenty-six (15.9%) patients died during the year following recovery from acute infection, and no relapse of listeriosis was observed. The main factors associated with 1-year early mortality were age (*p* = 0.007), diabetes mellitus (*p* = 0.033), cancer (*p* < 0.001) and metastasis (*p* < 0.001). Forty-seven (28.7%) patients died during the 5 years after recovery from listeriosis. Factors associated with 5-year mortality were age (*p* < 0.001), diabetes mellitus (*p* = 0.002), chronic kidney disease (*p* = 0.034), liver disease (*p* = 0.050), cancer (*p* < 0.001), metastasis (*p* < 0.001) or chemotherapeutic treatment (*p* < 0.021), and bacteraemia as clinical presentation of listeriosis (*p* = 0.013). The absence of comorbidities (*p* = 0.022) and pregnancy at presentation (*p* = 0.021) acted as protective factors for this outcome. Table 3 compares the 1-year mortality of patients after recovery from severe listeriosis with the 1-year mortality of the general population of Andalusia, according to the Spanish National Institute of Statistics (https://ine.es/). This table shows that our sub-population presents mortality rates more than ten times higher than in the general population for all age sub-groups.

**Table 2:** Main characteristics of the patients who recovered from acute infection (*n* = 164) stratified by 1-year and 5-year early mortality

Characteristic	Surviving patients after acute infection^1^	One-year early mortality	*P*-value^2^	Five-year early mortality	*P*-value^2^
Total	117 (71.3)	26 (15.9)		47 (28.7)	
Sex, *n* (%)			0.966		0.472
Women	52 (74.3)	11 (15.7)		18 (25.7)	
Men	65 (69.1)	15 (16.0)		29 (30.9)	
Age, x (sd)	56.9 (19.0)	69.2 (15.3)	0.007*	68.3 (15.2)	< 0.001*
Age, *n* (%)			0.083		0.013*
< 40 years	26 (92.9)	1 (3.6)		2 (7.1)	
41–50 years	18 (75.0)	3 (12.5)		6 (25.0)	
51–60 years	17 (77.3)	3 (13.6)		5 (22.7)	
61–70 years	21 (65.6)	5 (15.6)		11 (34.3)	
71–80 years	22 (71.0)	5 (16.1)		9 (29.0)	
> 80 years	13 (48.1)	9 (33.3)		13 (51.9)	
Year of diagnosis, *n* (%)			0.460		0.853
2005–2013	43 (70.5)	8 (13.1)		18 (29.5)	
2014–2021	74 (71.8)	18 (17.5)		29 (28.2)	
Comorbidities					
None	21 (91.3)	0 (0.0)	0.027*	2 (8.7)	0.022*
Diabetes mellitus	22 (52.4)	11 (26.2)	0.033*	20 (47.6)	0.002*
Hypertension	38 (67.9)	7 (12.5)	0.397	18 (32.1)	0.477
COPD	8 (72.7)	1 (9.1)	0.697	3 (27.3)	1.000
Asthma	5 (83.3)	1 (16.7)	1.000	1 (16.7)	0.675
OAHSD	4 (57.1)	3 (42.9)	0.080	3 (42.9)	0.674
Chronic kidney disease	9 (50.0)	5 (27.8)	0.169	9 (50.0)	0.034*
Heart failure	4 (57.1)	1 (14.3)	1.000	3 (42.9)	0.674
Ischaemic cardiopathy	11 (68.8)	4 (25.0)	0.472	5 (31.3)	1.000
Arrythmia	8 (61.5)	4 (30.8)	0.226	5 (31.5)	0.523
Valvopathy	2 (33.3)	2 (33.3)	0.242	4 (66.7)	0.057
Alcoholism	10 (76.9)	3 (23.1)	0.694	3 (23.1)	0.759
Hepatopathy	10 (52.6)	6 (31.6)	0.086	9 (47.4)	0.050*
Cancer	12 (36.4)	13 (39.4)	< 0.001*	21 (63.6)	< 0.001*
Metastasis	0 (0.0)	7 (87.5)	< 0.001*	8 (100.0)	< 0.001*
Chemotherapy	2 (28.6)	3 (42.9)	0.080	5 (71.4)	0.021*
HIV infection	3 (42.9)	3 (42.9)	0.080	4 (57.1)	0.194
Immunosuppression	17 (63.0)	6 (22.2)	0.322	10 (37.0)	0.292
Cognitive impairment	2 (40.0)	2 (40.0)	0.179	3 (60.0)	0.142
Morbid obesity	8 (72.7)	2 (18.2)	1.000	3 (27.3)	1.000
Autoimmune disease	12 (85.7)	1 (7.1)	0.474	2 (14.3)	0.243
Pregnancy	13 (100.0)	0 (0.0)	0.132	0 (0.0)	0.021*
Clinical presentation of listeriosis					
Meningitis	38 (76.0)	6 (12.0)	0.371	12 (24.0)	0.382
Meningoencephalitis	17 (81.0)	3 (14.3)	1.000	4 (19.0)	0.297
Rhombencephalitis	11 (68.8)	4 (25.0)	0.472	5 (31.3)	1.000
Sepsis	11 (78.6)	2 (14.3)	1.000	3 (21.4)	0.567
Bacteraemia	25 (56.8)	9 (20.5)	0.329	19 (43.2)	0.013*
Gastroenteritis	8 (80.0)	1 (10.0)	0.706	2 (20.0)	0.726
Device-associated infection	4 (66.7)	1 (16.7)	1.000	2 (33.3)	1.000
Other	3 (100.0)	0 (0.0)	1.000	0 (0.0)	0.558

*COPD* Chronic obstructive pulmonary disease, *HIV* Human immunodeficiency virus, *OAHSD* Obstructive apnoea–hypopnea sleep syndrome

^1^Percentage of surviving patients from the total patients of each variable (rows)

^2^*P*-value of the T test for quantitative variables (age), and of the chi-square test for qualitative variables or Fisher's exact test when the conditions for application of the chi-square test were not met

^*^p < 0.05

**Table 3:** One-year early mortality after resolution of listeriosis and annual mortality rate in Andalusia according to the most recent data (National Institute of Statistics, Spain, 2020)

Age group	One-year early mortality after recovery	One-year mortality rate in Andalusia for general population
	Rate^1^	Rate^1^
< 40 years	38.46	0.43
41–50 years	166.67	1.35
51–60 years	167.47	4.15
61–70 years	238.10	9.75
71–80 years	227.27	15.25
> 80 years	692.31	69.42

^1^Mortality rate is expressed in terms of number of deaths per 1000

Of the 47 patients who died within 5 years after recovery from infection, the causes were cancer in 22 (46.8%) cases, infection in 16 (34.0%) cases, and sequelae of listeriosis (i.e. neurolisteriosis) in 3 (6.4%) cases. Table 3 shows the results of the multivariate analysis performed for the 3 outcomes of interest.

**Fig. 3:**
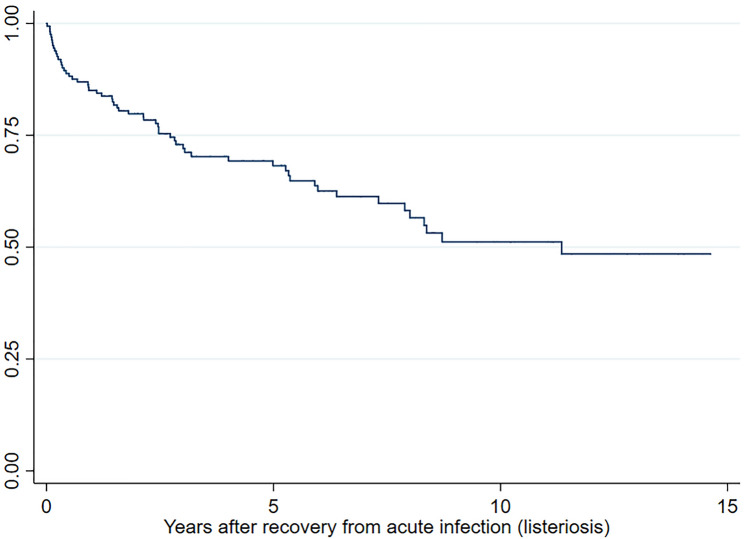
Kaplan–Meier survival estimates for the outcome death after recovery from acute listeriosis

**Fig. 4:**
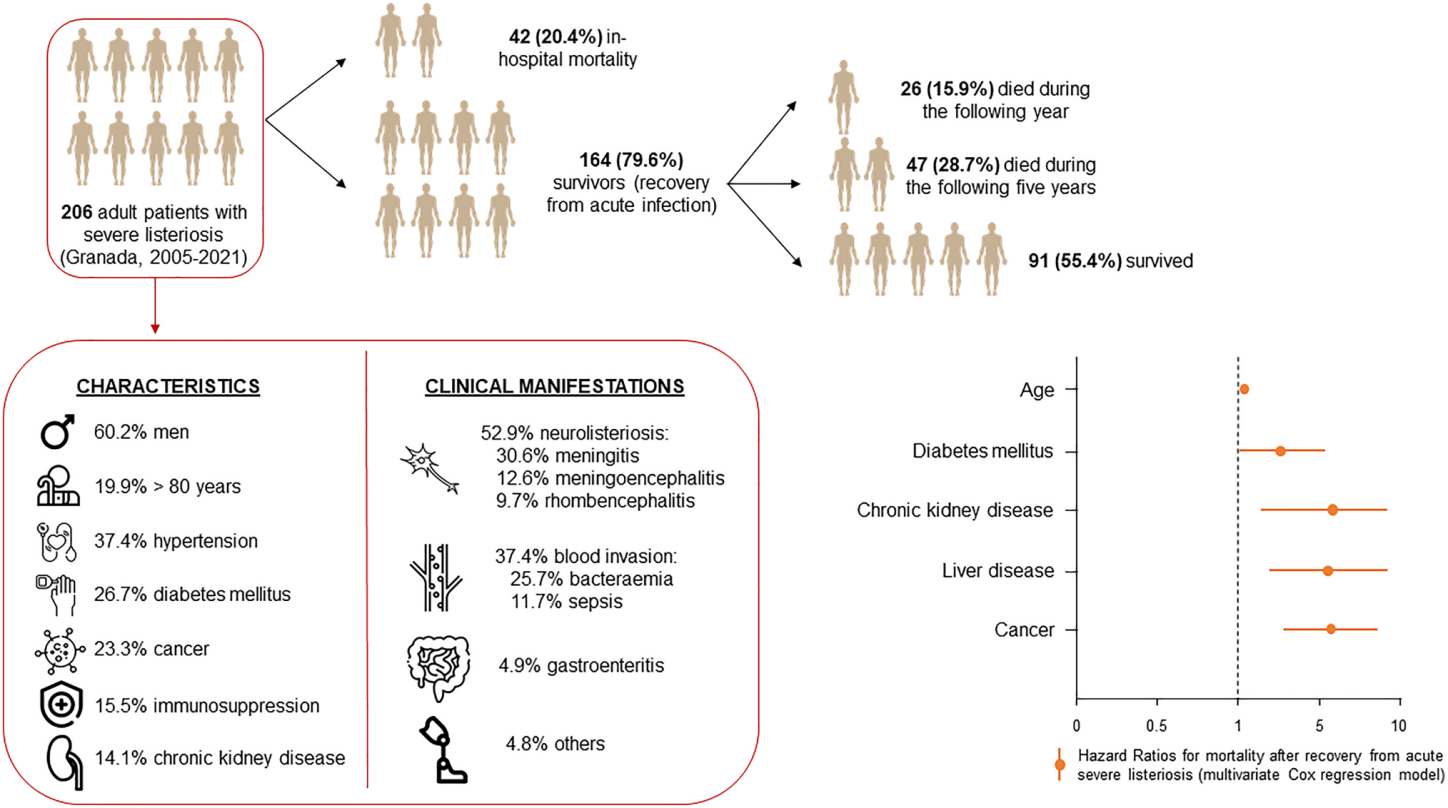
Main findings of the study

Regarding survival analysis, Fig. 3 shows the Kaplan–Meier estimates for the outcome death after recovery from listeriosis of the 164 patients that survived the acute infection. The median follow-up was 3 years (1,098 days). The median survival time (50% of deaths of the followed cohort) was reached at 11.3 (4,141 days) years of follow-up. Table 4 shows the results from multivariate Cox regression models. The factors that showed association with the time to death were age (HR: 1.03; 95% CI 1.02–1.07), diabetes mellitus (HR 1.86, 95% CI 1.01–3.44), chronic kidney disease (HR 3.96, 95% CI 1.87–8.38), liver disease (HR 3.62, 95% CI 1.64–8.51), cancer (HR 3.76, 95% CI 1.90–7.46), metastases (HR 12.88, 95%CI 3.61–45.90), after adjusting for all collected sociodemographic and clinical potential confounders. No association was found regarding the other comorbidities or the different clinical manifestations of listeriosis. Figure 4 summarises the main findings of this study.

**Table 4:** Multivariate analysis. Factors associated with in-hospital mortality, 1-year, and 5-year early mortality after resolution of listeriosis

Characteristic	In-hospital mortality	One-year early mortality after recovery	Five-year early mortality after recovery	Adjusted Cox regression models
	OR (IC 95%)^1^	OR (IC 95%)^1^	OR (IC 95%)^1^	HR (IC 95%)^2^
Sex (men)	1.58 (0.74–3.39)	0.77 (0.32–1.88)	0.97 (0.47–2.03)	1.06 (0.59–1.91)
Age	1.04 (1.02–1.07)*	1.07 (1.02–1.12)*	1.05 (1.02–1.08)*	1.04 (1.02–1.07)*
No comorbidities	0.24 (0.03–1.91)	0.12 (0.02–0.89)*	0.31 (0.07–1.45)	0.90 (0.19–4.24)
Diabetes mellitus	0.80 (0.37–1.75)	1.67 (0.65–4.28)	2.08 (1.03–3.42)*	1.86 (1.01–3.44)*
COPD	1.89 (0.64–5.58)	0.34 (0.04–2.93)	0.56 (0.13–2.37)	1.04 (0.42–2.57)
Chronic kidney disease	2.11 (0.87–5.14)	1.84 (0.56–6.00)	2.20 (0.77–6.24)	3.96 (1.87–8.38)*
Heart failure	3.37 (1.04–10.90)*	0.54 (0.06–4.86)	1.14 (0.23–5.60)	1.02 (0.28–3.67)
Liver disease	0.25 (0.03–1.94)	4.35 (2.33–12.18)*	3.61 (1.27–10.26)*	3.62 (1.54–8.51)*
Cancer	2.01 (0.93–4.35)	5.72 (2.23–14.66)*	4.16 (1.65–10.52)*	3.76 (1.90–7.46)*
Metastasis	3.27 (1.01–10.60)*	19.50 (4.94–49.63)*	6.60 (2.79–15.59)*	12.88 (3.61–45.90)*
Chemotherapy	2.72 (0.73–10.15)	1.42 (0.25–8.11)	1.88 (0.30–11.98)	0.55 (0.15–2.07)
Bacteraemia	0.74 (0.31–1.73)	1.52 (0.61–3.84)	2.69 (1.24–5.86)*	1.80 (0.97–3.34)
Sepsis	3.28 (1.26–8.54)*	0.96 (0.19–4.76)	0.71 (0.18–2.80)	0.36 (0.12–1.09)

*COPD* Chronic obstructive pulmonary disease

^1^Adjusted Odds Ratio of multivariate logistic regression models. Models are adjusted for age, sex, year of diagnosis,

comorbidities, and clinical presentation of listeriosis

^2^Adjusted Hazard Ratio of multivariate Cox regression models for the time to death after recovery of listeriosis in patients that survived acute infection

## Discussion

This longitudinal observational study of all reported cases of listeriosis in the province of Granada for 17 years shows a high in-hospital mortality (20.4%) and, more importantly, a high early mortality after recovery, reaching 15.9% 1 year after recovery from infection and 28.7% during the following 5 years. These data suggest that listeriosis could be a marker of early mortality. Furthermore, in our sample, we observed that adult patients who develop severe listeriosis tend to have a high frequency of comorbidities, especially cancer, immunosuppression, and chronic disorders, such as diabetes mellitus or hypertension, as well as advanced age. These data underline the special frailty of these patients and help to explain the early mortality observed. Finally, our study helps to identify the main factors associated with a fatal prognosis in adult patients with listeriosis, mainly advanced age, diabetes mellitus, chronic kidney disease, liver disease and cancer.

As previously described in the scientific literature, listeriosis is an opportunistic disease whose infection mainly targets vulnerable risk groups [12]. Thus, excluding vertical transmission to neonates, listeriosis particularly affects cancer patients, immunosuppressed patients, pregnant women and elderly patients [2]. These data fully coincide with the sociodemographic and clinical characteristics of our sample, in which only 11.7% showed no comorbidities according to their medical history.

Regarding in-hospital mortality, our data showed lower figures than those reported by Charlier et al. in France [3], who found 40% in-hospital mortality in cases of bacteraemia (including bacteraemia or sepsis) and 27% in cases of neurolisteriosis (including meningitis, meningoencephalitis or rhombencephalitis), compared to 25% and 20% in our cohort, respectively. Our data are similar to those reported by Thønnings et al. in Denmark [13], who found 23% mortality in cases of sepsis and 25% in cases of meningitis. In our study, sepsis was shown to be the main clinical form of listeriosis associated with in-hospital mortality. Interestingly, neurolisteriosis was not a worse prognostic risk factor per se in our sample, which contrasts with existing knowledge. Pregnant women in our cohort showed a lower risk of mortality in comparison with the rest of clinical manifestations recorded in our study. This might be partially explained by the younger age of this sub-population and the lower frequency of relevant comorbidities. The main associated comorbidities were advanced age, heart failure, renal disease, and metastasis. Although cancer and comorbidities have been noted as risk factors for in-hospital mortality elsewhere [3], the specific comorbidities that increase this risk are still unknown. Their detection could help to identify patients with listeriosis at higher risk on admission. In turn, this would help design preventive strategies aimed at improving their prognosis. In our study, we did not observe significant differences in mortality due to listeriosis over time (from 2005 to 2021), although there was a trend towards a reduction in these figures in more recent years. This trend might be partially explained by the significant improvement of the institutions involved in the study in terms of intensive care and hospitalisation resources in recent years. Finally, we also observed a higher frequency of in-hospital death in men.

Concerning early mortality after recovery from acute infection, the high percentage of patients who died during the year following recovery was striking, almost one in six. The 1-year death rate of our sample was strikingly higher than mortality rates of Andalusian general population for all age groups, although no data on patients with similar comorbidities were available to compare. These data suggest that listeriosis could be a clinical manifestation of more serious processes. It is evident that early mortality is higher in patients diagnosed with cancer, especially at metastatic stages. The association between listeriosis and cancer severity has already been reported in previous studies [14]. Nevertheless, in our study, we have also found an association with advanced age and liver disease (mainly previous diagnosis of chronic viral hepatitis). Our findings could suggest that it is necessary to intensify the immediate follow-up of elderly patients with previous liver conditions after recovery from listeriosis. In this same vein, follow-up consultations in primary care could help to early identify those patients at higher risk and their alarm symptoms, as has been done during the pandemic regarding the most severe cases of COVID-19 [15, 16]. Finally, the high percentage of patients whose cause of death was another infection was striking. These data reinforce that, preventive strategies, whether vaccines, pharmacological or lifestyle-related, aimed at strengthening the immune system, and improving access to health system (given that the early antibiotic treatment influences the prognosis of listeriosis), could help to improve the figures observed in our study.

Five years after recovery, the percentage of deaths increased to almost one in three patients. Although listeriosis particularly affects patients of advanced age and with previous conditions, in our opinion, this does not justify such a high early mortality as observed. Again, advanced age and the presence of cancer and metastases showed a very strong association in the adjusted models. In addition, previous liver disease (which proved to be one of the main indicators of mortality in our study) and bacteraemia as clinical presentation were associated with this outcome. When *L. monocytogenes* is able to reach the bloodstream, it is because it has overcome the natural immunological barriers of its host [17] and, therefore, this could be a marker of immunological weakness. Additionally, neurolisteriosis usually shows more identifiable clinical manifestations that facilitate its diagnosis and early treatment compared with bacteraemia. Nevertheless, the group of patients with central nervous system involvement showed a higher frequency of sequelae or persistent symptoms. This could be explained by factors, such as differential follow-up bias (i.e. these patients were systematically scheduled for follow-up programmes by the service of Neurology) or irreversible damage to specific functions of the central nervous system. In any case, no clinical manifestation showed association with mortality in Cox regression models.

Finally, given that follow-up period was different for each patient (e.g. patients followed from 2007 had more probability of developing the outcome than those followed since 2021), the multivariate Cox regression models helped to identify factors associated with time to death after recovery from acute listeriosis. Those factors independently associated with worse prognosis were age, diabetes mellitus, chronic kidney disease, liver disease and cancer, therefore conforming the most vulnerable group of patients after discharge. No specific clinical manifestation of listeriosis was identified as prognostic factor in our survival analyses.

The main strengths of our study lie in its multicentre nature (it included hospitals and notification centres across the province of Granada, through the Andalusian Epidemiological Surveillance System assessed within the Provincial Health Delegation of Granada), in the long period during which the registry was available (from 2005, the date of the first notified cases of listeriosis), and in the possibility to access medical records and collect detailed and reliable clinical information. However, although our data showed high early mortality after severe listeriosis, it is not possible to conclude that listeriosis itself is a marker of early mortality. For this assertion to be true, it would be necessary to compare these patients with a control group with similar characteristics in terms of vulnerability (age and presence of comorbidities); conversely, it can be concluded that listeriosis is strongly associated with poor health conditions that frequently lead to a fatal prognosis and, thus, improved follow-up and identification of potentially severe cases could be useful. To help design effective preventive strategies, future studies should analyse potential differences in the prognosis of patients with listeriosis compared to patients without listeriosis but with similar comorbidities. Another perceived limitation of our study was the inclusion of cases notified through our epidemiological system. It is known that the most frequent clinical manifestation of listeriosis is gastroenteritis, generally in the context of foodborne outbreaks [2]. However, most of these clinical infections go unnoticed and are not diagnosed by laboratory tests, making it impossible to identify them as confirmed cases and to notify them. Therefore, our selection of notified cases implies that our sample included the most severe cases of listeriosis, most of which correspond to those requiring hospitalisation (meningitis, sepsis, etc.). We did not include the time and type of antibiotic treatment during Emergency management of patients, as this information was poorly registered in the medical records before 2010. However, this information may constitute an important prognostic factor to consider in multivariate analyses. We tried to partially overcome this bias by adjusting for year of treatment and clinical presentation, assuming that antibiotic treatment was similar for these sub-groups of patients. Finally, it should be noted that, despite gathering all the data for a province and a period of more than 15 years, the sample size of our study is not large enough to obtain precise conclusions. Accordingly, the confidence intervals obtained from multivariate analyses are very wide and only in few cases allowed us to detect relevant differences. Therefore, our results should be interpreted cautiously. Nevertheless, we believe that the identification of associations through bivariate and even multivariate analysis, despite the aforementioned limitations, allowed us to make an interesting approach to potential factors involved in this early mortality after listeriosis.

In any case, our results should help to design prevention and public health strategies to improve the prognosis of patients with severe listeriosis after its resolution, as well as to improve follow-up consultations in neurology, infectious diseases, and primary care. Future studies in other settings are warranted to corroborate our results.

## Conclusion

Listeriosis is an infection that predominantly affects elderly, immunocompromised individuals, and patients with high-risk health conditions, such as cancer, chronic diseases, or pregnancy. In-hospital mortality due to this infection was very high in our cohort and was mainly associated with older age and with the presence of heart failure, metastatic cancer, and sepsis as clinical manifestation. In addition, our study highlights the high early mortality that occurs after resolution of the infection. Particularly, advanced age, the presence of cancer, previous liver disease, diabetes mellitus, and chronic kidney disease acted as risk factors for early mortality after recovery from acute listeriosis. It is necessary to increase knowledge about these risk factors to improve the prognosis of listeriosis after resolution of infection and to optimise individualised follow-up strategies.
